# Candidate genetic analysis of plasma high-density lipoprotein-cholesterol and severity of coronary atherosclerosis

**DOI:** 10.1186/1471-2350-10-111

**Published:** 2009-10-30

**Authors:** Suet Nee Chen, Mehmet Cilingiroglu, Josh Todd, Raffaella Lombardi, James T Willerson, Antonio M Gotto, Christie M Ballantyne, AJ Marian

**Affiliations:** 1 Center for Cardiovascular Genetics, Brown Foundation Institute of Molecular Medicine, The University of Texas Health Science Center and Texas Heart Institute, Houston, TX, USA; 2 Graduate Program in Cardiovascular Sciences, Baylor College of Medicine, Houston, TX, USA; 3 University of Cincinnati, Division of Cardiology, Cincinnati, OH, USA; 4 Section of Cardiology, University of North Carolina, Chapel Hill, NC, USA; 5 Weil College of Medicine of Cornel University, New York, NY, USA; 6 Section of Atherosclerosis and Vascular Medicine, Department of Medicine, Baylor College of Medicine, Houston, TX, USA; 7 Methodist DeBakey Heart and Vascular Center, Houston TX, USA

## Abstract

**Background:**

Plasma level of high-density lipoprotein-cholesterol (HDL-C), a heritable trait, is an important determinant of susceptibility to atherosclerosis. Non-synonymous and regulatory single nucleotide polymorphisms (SNPs) in genes implicated in HDL-C synthesis and metabolism are likely to influence plasma HDL-C, apolipoprotein A-I (apo A-I) levels and severity of coronary atherosclerosis.

**Methods:**

We genotyped 784 unrelated Caucasian individuals from two sets of populations (Lipoprotein and Coronary Atherosclerosis Study- LCAS, N = 333 and TexGen, N = 451) for 94 SNPs in 42 candidate genes by 5' nuclease assays. We tested the distribution of the phenotypes by the Shapiro-Wilk normality test. We used Box-Cox regression to analyze associations of the non-normally distributed phenotypes (plasma HDL-C and apo A-I levels) with the genotypes. We included sex, age, body mass index (BMI), diabetes mellitus (DM), and cigarette smoking as covariates. We calculated the q values as indicators of the false positive discovery rate (FDR).

**Results:**

Plasma HDL-C levels were associated with sex (higher in females), BMI (inversely), smoking (lower in smokers), DM (lower in those with DM) and SNPs in *APOA5, APOC2*, *CETP, LPL *and *LIPC *(each q ≤0.01). Likewise, plasma apo A-I levels, available in the LCAS subset, were associated with SNPs in *CETP*, *APOA5*, and *APOC2 *as well as with BMI, sex and age (all q values ≤0.03). The *APOA5 *variant S19W was also associated with minimal lumen diameter (MLD) of coronary atherosclerotic lesions, a quantitative index of severity of coronary atherosclerosis (q = 0.018); mean number of coronary artery occlusions (p = 0.034) at the baseline and progression of coronary atherosclerosis, as indicated by the loss of MLD.

**Conclusion:**

Putatively functional variants of *APOA2*, *APOA5, APOC2*, *CETP, LPL*, *LIPC *and *SOAT2 *are independent genetic determinants of plasma HDL-C levels. The non-synonymous S19W SNP in *APOA5 *is also an independent determinant of plasma apo A-I level, severity of coronary atherosclerosis and its progression.

## Background

Coronary artery disease is the most common cause of death in the western hemisphere and is expected to become the leading cause of morbidity and mortality in the world by the year 2020 [1]. Plasma high-density lipoprotein-cholesterol (HDL-C) level is a major determinant of susceptibility to coronary atherosclerosis in the general population [2-7]. A low plasma HDL-C level is the most common lipid abnormality found in families with premature coronary atherosclerosis [2]. Plasma HDL-C level is inversely associated with the progression of coronary atherosclerosis and cardiovascular mortality [3-7]. In contrast, raising the plasma high HDL-C level, even modestly, with the exception of inhibition of cholesteryl ester transfer protein (CETP) with torcetrapib, protects against atherosclerosis and reduces adverse cardiovascular events [4,8-10].

Genetic studies of Mendelian traits have firmly established the impact of genetic mutations on plasma HDL-C levels. A prototypic example is Tangier disease, wherein mutations in *ABCA1 *lead to very low plasma HDL-C and apolipoprotein (apo) A-I levels and premature coronary atherosclerosis [11,12]. Plasma HDL-C level in the general population is also a heritable trait. Heritability of plasma HDL-C level has been estimated to be greater than 50% in most studies [13-18]. The estimates of heritability in the Strong Heart Family Study and HERITAGE family study were 50% and 52%, respectively [14,15]. Unlike the Mendelian disorders, however, the specific genetic variants that contribute to plasma HDL-C levels in the general population are largely unknown. The advent of genome-wide association studies (GWAS) has raised considerable interest in identifying novel genetic determinants of complex traits, such as plasma HDL-C. Accordingly, several genes that influence plasma HDL-C levels have been identified [19,20]. Collectively, the associated variants in GWAS accounted for only 5-8% of the variation in the plasma HDL-C levels [19,20]. Hence, much of the heritability of plasma HDL-C levels has remained unexplained.

We genotyped 784 unrelated Caucasian individuals from two independent populations of Lipoprotein and Coronary Atherosclerosis Study (N = 333) and TexGen (N = 451) for 94 non-synonymous or regulatory single nucleotide polymorphisms (SNPs) in 42 genes implicated in HDL-C biosynthesis and metabolism. We analyzed association of the SNPs with plasma HDL-C and apo A-I levels as well as with the severity and progression of coronary atherosclerosis.

## Methods

### Study population

All participants signed informed consent for the genetic studies and the institutional review board approved the study. We include 784 unrelated Caucasians, comprised of the LCAS subpopulation (N = 333) and the TexGen subpopulation (N = 451). The study design and the main results of the LCAS have been published [21]. In brief, the main LCAS included 372 Caucasians who had at least 1 coronary lesion causing 30% to 75% luminal diameter stenosis on quantitative coronary angiography and had a plasma low-density lipoprotein-cholesterol (LDL-C) level of 115 to 190 mg/dl despite diet. DNA samples from 333 Caucasians were available. The baseline values of plasma total cholesterol, LDL-C, HDL-C, triglyceride (TG) and apolipoprotein levels were measured in all participants at the baseline. Quantitative indices of severity of coronary atherosclerosis were measured in 289 participants.

Given the relatively small sample size of the LCAS population, we recruited 451 patients from outpatient clinics (the TexGen study). The TexGen subpopulation was matched for age and the mean plasma HDL-C level to the LCAS subpopulation. Because of the small number of females in the LCAS population, we included relatively more females in the TexGen subpopulation. Individuals taking medications with direct effects on plasma HDL-C levels, such as niacin and fibrates were not included in the study. Those with advanced co-morbid conditions, including malignancies, advanced heart failure or valvular heart disease were not included. Demographic data were collected and fasting plasma total cholesterol, HDL-C, LDL-C and TG levels were measured in all TexGen participants.

### Selection of the candidate genes and SNPs

We selected the candidate genes based on *a prior *knowledge of their involvement in pathways leading to synthesis, maturation, or catabolism of HDL-C in humans or in experimental animals. Upon selection of the candidate genes, we searched the SNP database (dbSNP Build 129) to identify non-synonymous SNPs and the regulatory SNPs in the 5' or 3' regions of the genes of interest. We included only SNPs that have been validated, have a known minor allele frequency (MAF) in the database or were previously shown to be associated with plasma HDL-C levels. Given the size of the study population, we mostly selected the SNPs that had known minor allele frequencies (MAFs) of > 0.1, while realizing that the MAFs could differ in our study population. In view of the presence of a very large number of SNP in each gene locus, the selected SNPs comprised only a fraction of the total SNPs in the candidate genes loci. We genotyped the selected SNPs by fluorogenic 5' nucleotidase (Taqman) assays using an ABI PRISM^® ^7900HT Real-Time PCR instrument. The PCR conditions were 1 cycle at 95°C for 10 min, followed by 40 cycles at 92°C for 15 sec and 60°C for 1 min as recommended by the manufacturer (Applied Biosystems, Foster City, CA). Investigators who had no knowledge of the angiographic and clinical data performed the genotyping.

Since there was no known candidate SNP in *ATP5B*, a high affinity HDL-C receptor for apo A-I [22], we sequenced its entire coding region, 3' untranslated sequence and a 2 kbp 5' genomic fragment in 50 randomly selected individuals from the LCAS subpopulation. We performed the sequencing using the Big Dye Terminator Cycle Sequencing Ready Reaction Kit on an ABI Genetic Analyzers in sense and anti-sense directions. The sequences were analyzed and "Blasted" against the genome databases.

### Statistical analysis

We expressed the parametric variables as mean ± SD and tested the variables for Gaussian distribution by the Shapiro-Wilk normality test. We tested the distribution of genotypes of each SNP for departure of the Hardy-Weinberg equilibrium (HWE) by the X^2 ^test using a web-based program [23]. We compared the mean values between the two groups by the t test and analyzed the non-parametric variables by the Fisher exact test. We assigned indicators to genotypes as 0, 1 and 2 to represent 0, 1 and 2 copies of the common allele (additive genetic model). Likewise, we assigned indicators 0 and 1 to females and males, respectively. Since the distributions of plasma HDL-C and apo A-I levels deviated significantly from normality, we analyzed associations between the predictors and plasma HDL-C and apo A-I levels by Box-Cox regression analysis using likelihood ratio tests (16,000 iterations). We analyzed each potential predictor separately and included those with p values ≤ 0.10 in the multivariate Box-Cox regression analysis to determine independent association of the predictors with the phenotypes. We calculated the False positive Discovery Rate (FDR) for the results of multivariate analysis by calculating the q values by the method of Storey and Tibshirani [24]. All statistical analyses were performed using STATA, IC 10.1 for Macintosh.

## Results

### Characteristics of the study population

The baseline characteristics of the study population including the characteristics of the LCAS and TexGen subpopulations are shown in Table 1. The subpopulations were matched for age and mean plasma HDL-C levels. There were more females and more individuals with diabetes mellitus in the TexGen subpopulation. There were modest albeit statistically significant differences in body weight (~3.4 Kg), body mass index (BMI, ~1.2 Kg/m^2^), mean systolic or diastolic blood pressure (1-3 mmHg) and the history of myocardial infarction between the two subpopulations (Table 1).

**Table 1:** Baseline Characteristic of the Study Population and Subpopulations

	**All**	**LCAS**	**TexGen**	**p**
**N**	784	333	451	---
**Demographics**				
Age (years)	59.3 ± 11.0	59.3 ± 7.7	59.2 ± 13.2	0.527
Male/Female (%)	586/198 (75/25)	281/52 (84/16)	305/146 (68/32)	<0.001
Height (m)	1.73 ± 0.09	1.74 ± 0.08	1.73 ± 0.10	0.328
Body weight (Kg)	86.8 ± 18.8	84.9 ± 15.6	88.3 ± 20.7	0.006
Body mass index (Kg/m^2^)	28.8 ± 5.5	28.1 ± 4.5	29.3 ± 6.1	0.001
Systolic blood pressure (mmHg)	125.9 ± 19.3	124.5 ± 15.3	127.1 ± 21.8	0.029
Diastolic blood Pressure (mmHg)	75.8 ± 11.9	76.7 ± 8.8	75.1 ± 13.7	0.028
Diabetes mellitus (%)	117 (14.5)	11 (3.3)	106 (23.5)	<0.001
Smoker (%)	137 (17.5)	68 (20)	69 (15)	0.071
History of myocardial infarction (%)	289 (36.9)	131 (39)	158 (35)	<0.001
**Plasma Lipids levels**				
TC (mg/dl)	200.1 ± 56.5	220.0 ± 24.5	185.2 ± 68.0	<0.001
HDL-C (mg/dl)	43.8 ± 13.9	43.8 ± 11.3	43.8 ± 15.5	0.671
LDL-C (mg/dl)	123.9 ± 46.9	143.9 ± 19.9	107.5 ± 55.1	<0.001
LDL-C/HDL-C	3.02 ± 1.28	3.49 ± 0.91	2.65 ± 1.40	<0.001
Triglyceride (mg/dl)	169.7 ± 154.8	161.5 ± 57.1	175.8 ± 198.0	0.101
**Plasma apolipoprotein levels (LCAS only)**				
**N**		333		
Apolipoprotein A-I		132.7 ± 27.4		
Apolioprotein B		134.4 ± 20.8		
Apolipoprotein B/Apolioprotein A-I		1.05 ± 0.26		
Apolipoprotein C-III		35.2 ± 32.8		
**Quantitative indices of severity of coronary atherolosclerosis (LCAS only)**				
N		289		
Average baseline MLD (mm)		1.68 ± 0.40		
Mean number of coronary lesions		2.99 ± 1.38		
Number of subject with ≥ two coronary lesion (%)		241 (83%)		
Mean number of coronary occlusions		0.29 ± 0.55		
Number of subject with ≥ one coronary		72 (25%)		

**Abbreviations**: m: meter; Kg: Kilogram; mmHg: millimeter mercury; mg/dl: milligram per deciliter; TC: Total cholesterol; HDL-C: High-density lipoprotein-cholesterol; LDL-C: low-density lipoprotein-cholesterol; MLD: minimal lumen diameter.

As would be expected, mean plasma HDL-C levels were similar between the two groups. However, plasma total cholesterol and LDL-C levels were significantly higher in the LCAS subpopulations, as a high plasma LDL-C level was a requirement for inclusion in the LCAS. Plasma apolipoprotein levels and quantitative indices of severity of coronary atherosclerosis were available only in the LCAS subpopulation (Table 1).

### Candidate genes and SNPs

The list of biologically plausible candidate genes and SNPs analyzed in the present study and the MAFs in the entire population are shown in Additional File 1. The genotypes of all but 3 SNPs followed the HWE. The genotypes of SNPs rs4301822 in *APOF*, rs2306985 in *MTP *and rs2038068 in *PPARD *showed significant departure from the HWE (p < 0.05). The departure may reflect genotyping errors or the population genetic structure. Therefore, they were not included in the subsequent association analyses.

### Independent predictors of plasma HDL-C levels

Plasma HDL-C and apo A-I deviated significantly from the normal Gaussian distributions (z = 8.473, p < 0.00001 for HDL-C and z = 6.386, p < 0.00001). Likewise, the log10 transformed HDL-C levels were non-normally distributed (z = 2.360, p = 0.00913). Therefore, Box-Cox regression analysis was used to determine association of the potential predictors with plasma HDL-C and apo A-I levels. Sex, body mass index (BMI), diabetes mellitus (DM) and cigarette smoking were independent predictors of plasma HDL-C and apoA-I levels (Tables 2 and 3). The FDR for each of the observed associations of sex, BMI, DM and smoking all were very low (less than 0.1%). Therefore, they were included in the subsequent analyses as covariates.

**Table 2:** Independent Predictors of Median Plasma HDL-C Levels Excluding BMI and DM as Co-variates (N = 784)

**Predictors**		**Coefficient**	**X**^**2**^	**p**	**Q**
Sex (Female = 0, Male = 1)		-0.271	91.873	<0.001	<0.001
Smoking (Yes = 1, No = 0)		-0.118	14.432	<0.001	<0.001
**Gene**	**SNP**	**Coefficient**	**X**^2^	**p**	**Q**
*CETP*	rs12149545	0.082	20.727	<0.001	<0.001
*LPL*	rs328	0.094	10.870	0.001	0.004
*APOA5*	rs3135506	-0.145	8.729	0.003	0.008
*APOA2*	rs3829793	.213	8.259	0.004	0.009
	rs3813627	-0.202	7.207	0.007	0.013
*LIPC*	rs36041167	-0.088	5.965	0.015	0.024
*APOC2*	rs228911	0.041	5.544	0.019	0.026
*SOAT2*	rs2272296	0.055	4.538	0.033	0.040

**Abbreviations**: as in Table 2; SOAT2: Sterol O-acyltransferase 2

**Table 3:** Independent Predictors of Median Plasma apo A-I Levels (N = 333)

**Predictors**		**Coefficient**	**X**^**2**^	**P**	**q**
Sex (Female = 0, Male = 1)		-0.0271	79.986	<0.001	<0.001
BMI (Kg/m^2^)		-0.0012	27.609	<0.001	<0.001
Age (years)		0.0005	15.116	<0.001	<0.001
**Gene**	**SNP**	**Coefficient**	**X**^**2**^	**P**	**q**
*MTP*	rs17029215	0.0045	10.655	0.001	0.005
*CETP*	rs12149545	0.0042	7.852	0.005	0.018
	rs58821	0.0035	4.525	0.033	0.066
*APOC2*	rs228911	0.0036	5.875	0.015	0.034
*APOA5*	rs3135506	-0.0118	6.797	0.009	0.027
*SCARB1*	rs8388843	0.0040	6.497	0.011	0.028

**Abbreviations**: as in the previous Tables

We genotyped the study population for 94 SNPs in 42 genes (Additional File 1). Three SNPs were very rare and 2 not detected in the LCAS subpopulation. Therefore, we analyzed 89 SNPs in the entire population and included any potentially significance association, defined as p < 0.05, in the multivariate analysis. SNPs in *APOA5*, *APOC2*, *CETP*, *LIPC *and *LPL *were independent predictors of plasma HDL-C levels (Table 4). The FDR for each of the above genes was ≤ 1 percent. Considering that plasma HDL-C, DM and BMI may share common genetic etiology as part of the metabolic syndrome; we repeated the Box-Cox regression analysis after removal of BMI and DM from the analysis as covariates. The results, shown in Table 2, identify *APOA2 *and *SOAT2 *variants, in addition to SNPs in *APOA5*, *APOC2*, *CETP*, *LIPC *and *LPL *also as independent determinants of plasma HDL-C levels.

**Table 4:** Independent Predictors of Median Plasma HDL-C Levels (N = 784)

**Predictors**		**Coefficient**	**X**^**2**^	**p**	**Q**
Sex (Female = 0, Male = 1)		-0.327	102.294	<0.001	<0.001
BMI (Kg/m^2^)		-0.015	34.576	<0.001	<0.001
DM (Yes = 1, No = 0)		-0.224	33.335	<0.001	<0.001
Smoking (Yes = 1, No = 0)		-0.153	17.824	<0.001	<0.001
**Gene**	**SNP**	**Coefficient**	**X**^**2**^	**p**	**Q**
*CETP*	rs12149545	0.121	11.566	0.001	0.003
*LPL*	rs328	0.101	9.903	0.002	0.005
*APOC2*	rs228911	0.057	8.239	0.004	0.008
*APOA5*	rs3135506	-0.150	7.206	0.007	0.012
*LIPC*	rs36041167	-0.110	7.144	0.008	0.012

**Abbreviations**: BMI: Body mass index; DM: Diabetes mellitus. CETP: Cholesteryl Ester Transfer; LPL: Lipoprotein Lipase; APOC2: Apolipoprotein C-II; APOA5: Apolipoprotein A-V; LIPC: Hepatic Lipase

We also determined independent association of SNPs with plasma HDL-C levels as a non-parametric phenotype of less or greater than the median value of 43 mg/dl by logistic regression analysis (robust method). The results were largely concordant with the results of Box-Cox regression analysis, as sex, BMI, DM and smoking as well as SNPs in *APOA5*, *CETP *and *LPL *were independent predictors of plasma HDL-C levels (all q values ≤ 0.06).

Since plasma triglyceride and HDL-C levels may share common genetic determinants, we analyzed the association of plasma triglyceride levels with the demographic variables and SNPs in *APOA5*, *APOC2*, *CETP*, *LIPC *and *LPL*. We used Box-Cox regression for the analysis because of non-normal distribution of plasma triglyceride levels. The main determinants of plasma triglyceride levels in the present study population were BMI and *LPL *variants, while age and *APOA5 *had modest effects (Data not presented).

### Independent predictors of plasma apo A-I levels

Plasma apo A-I levels also did not follow a normal distribution. Therefore, we tested the association of plasma apo A-I levels with demographic predictors and SNP by Box-Cox regression. Sex (higher in females), BMI (inversely), age (positively) and SNPs in *MTP*, *CETP*, *APOC2*, *APOA5 *and *SCARB1 *were independent predictors of plasma apo A-I levels (Table 3).

### SNPs and severity of coronary atherosclerosis

There was no significant association between the demographic variables and the severity of coronary atherosclerosis at the baseline. However, baseline MLD was associated with the rs3135506 (S19W) SNP in *APOA5*. Those with the uncommon allele (GA only, no AA) had more severe coronary atherosclerosis than those with the common genotype (GG). The mean MLD was 1.42 ± 0.43 mm in those with the GA genotype (N = 11) and 1.69 ± 0.69 (N = 278) in those with the GG genotype (t = 2.204, p = 0.014, q = 0.021). Likewise, the number of coronary occlusion was greater in those with the GA as compared to those with the GG genotype (0.064 ± 0.67 vs. 0.28 ± 0.54, respectively, p = 0.034). It merits noting that the observed association with the severity of coronary artery disease was concordant with the effects of the SNP on plasma HDL-C (~6 mg/dl lower in those with GA genotype) and apo A-I (~13 mg/dl lower in those with the GA genotype) levels. The *MTP *variants (rs2306986) were also associated with the MLD at the baseline. The mean MLD was 1.66 ± 0.40 mm in individuals with the GG genotype (N = 273) as compared to 1.94 ± 0.34 mm in those with the GC genotype (N = 16, t = -2.8084, p = 0.0027, q = 0.008).

### SNPs and progression of coronary atherosclerosis

Plasma levels of HDL-C (regression coefficient: 0.004, 95% CI: [0.002-0.006], t = 3.36, p = 0.001) and treatment with fluvastatin (regression coefficient: 0.084, 95% CI: [0.032-0.136], t = 3.19, p = 0.002) were independent predictors of the progression of coronary atherosclerosis (change in MLD) in the LCAS population [21]. Therefore, we included HDL-C levels and treatment with fluvastatin as covariates in the genetic analysis. Progression of coronary atherosclerosis as determined by the change in MLD during the 2.5 years follow up, was associated with S19W SNP in *APOA5*. Those with the uncommon genotype (AG) had greater loss of MLD (0.25 ± 0.23 mm) over the follow up period than those with the common genotype (-0.05 ± 0.25 mm, p = 0.0048).

## Discussion

We analyzed association of plasma HDL-C levels with 89 putatively functional SNPs in 42 biologically candidate genes in a well-characterized Caucasian population. The results show that SNPs in genes encoding apo A-II (*APOA2*) apo A-V (*APOA5*), apo C-II (*APOC2*), cholesteryl ester transfer protein (*CETP*), hepatic lipase (*LIPC*), lipoprotein lipase (*LPL*) and Sterol O-acyltransferase 2 (*SOAT2*) as well as demographic variables sex, BMI, DM and smoking were independent predictors of plasma HDL-C levels. The false positive discovery rates (q values) for the observed associations were less than 5%, which is considered low. Consistent with the above, SNPs in *CETP*, *APOC2 *and *APOA5 *were also independent predictors of plasma apo A-I levels. Likewise, the *APOA5 *variant was also associated with the severity of coronary atherosclerosis at the baseline as well as progression of coronary atherosclerosis over a 2.5 years period.

The results of the present study are largely in accord with the known biological effects of the genes in HDL-C biosynthesis, maturation and catabolism and largely supported by the previous data for SNPs in *APOA5, CETP, LIPC *and *LPL *genes [20,25-31]. An interesting finding was the concordant association of the S19W SNP in *APOA5 *with plasma HDL-C and apo A-I levels as well as the severity of coronary atherosclerosis and its progression. *APOA5 *encodes apo A-V, which is a component of HDL-C and an important determinant of plasma triglyceride levels. The *APOA5 *S19W variant has been previously associated with hypertriglyceridemia as well as with plasma HDL-C levels [19,32]. However, the findings regarding the associations of the S19W SNP with the severity and progression of coronary atherosclerosis are new and in harmony with the effects of the *APOA5 *variants on plasma HDL-C, apo A-I and triglyceride levels. Thus, the findings further substantiate the clinical significance of *APOA5 *variants in coronary atherosclerosis.

The observed associations of plasma HDL-C and apo A-I levels with *APOC2 *variants are also novel, albeit intrinsic to the candidate gene approach is *a prior *biological plausibility of the candidates and hence, the findings were not unexpected. Apo C-II is an activator of lipoprotein lipase (LPL) and a regulator of plasma triglyceride levels. Loss-of-function mutations in *APOC2 *and *LPL *are associated with severe hypertriglyceridemia [33]. As in the present study, variants of *LPL *have been previously associated with plasma HDL-C levels [34].

Association studies that include multiple SNPs have an inherent risk of spurious associations. To shed some light on the possibility of association by chance alone, we calculated the FDR. We note the FDR for the above associations between plasma HDL-C and SNPs were quite low (q values of ≤ 0.01). Equally striking is the absence of significant associations between plasma HDL-C levels and SNPs in many of the selected genes that have been previously implicated - through studies in humans or animals - in HDL-C biosynthesis and metabolism. Various factors may contribute to the null results for the above genes including the characteristics of the study population, competing factors (demographic predictors), and conservative approach to analysis as well as the possibility of type II statistical error. The latter is typically due to interplay between the sample size of the study population, frequencies of the alleles and the effect sizes. Despite our *a prior *focus on common SNPs, the MAFs of 18 SNPs in our study population were <0. 05. Unless the uncommon alleles impart major effects on the plasma HDL-C levels, detection of a significant association would require a much larger population. Nevertheless, at an MAF of 0.05, assuming Hard-Weinberg equilibrium, the sample size of the study population provided 85% power to detect a 5 mg/dl difference in the mean plasma HDL-C levels between the common and uncommon genotypes, when α is set at 0.05. A larger sample size would be required to detect a smaller effect or the effects of even less frequent alleles. Likewise, we note that the secondary findings in the present study, namely, the observed associations between SNPs and the quantitative indices of severity of coronary atherosclerosis merit testing for replications. Finally, the study population was comprised of self-described Caucasians, which may not represent a genetically homogenous population. Hence, the results may be subject to possible population stratification.

The study is by no means comprehensive of all known genes and SNPs with possible roles in the HDL-C biosynthesis, maturation, conversion and/or catabolism. Recently SNPs in *GALNT2*, which encodes N-actetylgalactosaminyltransferase 2 locus were shown to be associated with plasma HDL-C and triglyceride levels in GWAS [19,35]. Similarly, SNPs at the *MMAB/MVK *locus, which encode mevalonate kinase and methylmalonic aciduria cblB type, respectively have also been associated with plasma HDL-C levels [20]. SNPs in *VNN1 *encoding pantetheinase or vannin 1 have also been associated with plasma HDL-C levels [36]. Additional genes in animal models also have been associated with plasma HDL-C levels, such as Sirt1, encoding sirtuin 1 [37]. We did not include these new genes primarily because their functional roles in HDL-C biosynthesis and metabolism have not yet been determined.

## Conclusion

We genotyped 784 Caucasian individuals for 94 candidate functional SNPs in 42 biologically plausible candidate genes implicated in HDL-C biosynthesis and metabolism. We report that SNPs in *APOA2*, *APOA5*, *APOC2*, *CETP*, *LIPC *and *LPL *are independent determinants of plasma HDL-C levels. SNPs in APOA5 are also associated with plasma apo A-I level, severity of coronary atherosclerosis as well as its progression.

## Competing interests

The authors declare that they have no competing interests.

## Authors' contributions

SNC carried out genotyping and assisted in data analysis, MC performed DNA sequencing and genotyping, JT carried out genotyping, RL also carried out genotyping and participated in phenotyping, JTW conceived and coordinated the TexGen study and recruited patients, AMG conceived, designed and coordinated the Lipoprotein and Coronary Atherosclerosis Study, CMB participated in the design of the study and phenotyping of the Lipoprotein and Coronary Atherosclerosis Study population and AJM conceived the genetic studies, analyzed the data and wrote the manuscript.

All authors read and approved the final manuscript.

## Pre-publication history

The pre-publication history for this paper can be accessed here:



## Supplementary Material

Additional file 1 **List of Candidate Genes and Putative Functional SNPs.** Click here for file
