# Supplementary material for: Candidate genetic analysis of plasma high-density lipoprotein-cholesterol and severity of coronary atherosclerosis
Source: BMC Med Genet. 2009 Oct 30;10:111. doi: 10.1186/1471-2350-10-111 (PMC2775733; doi:10.1186/1471-2350-10-111)
Supplement: Additional file 1 — List of Candidate Genes and Putative Functional SNPs. [file 1471-2350-10-111-S1.pdf]

Additional File 1

List of Candidate Genes and Putative Functional SNPs

Gene	Symbol	SNP ID	SNP	*MAF*
ATP-binding Cassette, sub-family A, member 1	*ABCA1*	rs2980083	-940T>G	0.489
		rs2422493	-477C>T	0.466
		rs2246293	-320G>C	0.460
		rs1800976	-191G>C	0.461
		rs2230806	R219K (G>A)	0.273
		rs4149313	I883M (A>G)	0.132
		rs2230808	R1587K (G>A)	0.232
ATP-binding cassette, sub-family B, member 1	*ABCB1*	rs9282564	N21D (A>G)	0.080
ATP-binding cassette, sub-family G, member 2	*ABCG2*	rs2231142	Q141K (C>A)	0.114
ATP-binding cassette, sub-family G, member 5 (sterolin 1)	*ABCG5*	rs6720173	Q604E (C>G)	0.167
ATP-binding cassette, sub-family G, member 8 (sterolin 2)	*ABCG8*	rs4148211	Y54C (A>G)	0.344
		rs4148217	T400K (C>A)	0.179
		rs6544718	A632V (T>C)	0.231
Angiopoietin-like 4	*ANGTL4*	rs1044250	T228M (C>T)	0.311
		rs35061979	R240Q (G>A)	ND
Apolipoprotein A-I	*APOA1*	rs670	-75G>A	0.179
Apolipoprotein A-II	*APOA2*	rs3813627	-1957C>G	0.338
		rs3829793	-1209G>T	0.333
		rs5082	-492A>G	0.379
Apolipoprotein A-IV	*APOA4*	rs675	T367S (A>T)	0.190
		rs5104	S147N (G>A)	0.122
Apolipoprotein A-V	*APOA5*	rs1729411	-3659C>T	0.135
		rs3135506	S19W (C>G)	0.029
Apolipoprotein C-II	*APOC2*	rs2288911	-42A>G	0.497
		rs5126	K77Q (A>C)	ND
Apolipoprotein C-III	*APOC3*	rs2854117	-482C>T	0.254
		rs2854116	-455T>C	0.356
		rs5130	3238T>C(3'UTR)	0.135
Apolipoprotein D	*APOD*	rs7659	1042T>C (3'UTR)	0.323
Apolipoprotein F	*APOF*	rs4301822	I311T (T>C)	0.008

ATP synthase, H+ transporting,mitochondrial F1 complex,β polypeptide	*ATP5B*	Novel	N257S (A>G)	0.004
Cholesteryl Ester Transfer Protein	*CETP*	rs12149545	-2708G>A	0.305
		rs4783961	-971A>G	0.497
		rs708272	-279G>A	0.380
		rs5882	I405V (A>G)	0.451
Clusterin	*CLU*	rs9331936	N317H (A>C)	0.003
		rs10503814	2718C>T(3'UTR)	0.040
Cubilin (intrinsic factor cobalamin receptor)	*CUBN*	rs1801222	S253F (C>T)	0.356 -
		rs1801224	T389P (A>C)	0.335
		rs1801231	S1559P (T>C)	0.103
		rs2271462	S1840G (G>A)	0.020
		rs3740168	R2575P (G>C)	0.020
		rs1801239	V2984I (A>G)	0.098
Fatty acid binding protein 2, intestinal	*FABP2*	rs1799883	A55T (G>A)	0.269
High density lipoprotein binding protein (vigilin)	*HDLBP*	rs7578199	N418S (A>G)	0.259
Klotho	*KL*	rs9536314	F352V (T>G)	0.143
Lecithin-cholesterol acyltransferase	*LCAT*	rs1134760	R173H (C>T)	0.182
Hepatic Lipase	*LIPC*	rs36041167	-592G>A	0.060
		rs1800588	-514C>T	0.093
		rs6083	N215S (A>G)	0.378
		rs207089	3'UTR (C>T)	0.226
Endothelial Lipase	*LIPG*	rs2000813	T111I (C>T)	0.302
		rs4939585	22607C>T(3'UTR)	0.362
Lipoprotein Lipase	*LPL*	rs1801177	D9N (G>A)	0.021
		rs268	N291S (A>G)	0.018
		rs328	S447T (C>G)	0.103
Myeloperoxidase	*MPO*	rs7208693	V53F (C>A)	0.029
		rs2759	V717I (A>G)	0.010
Microsomal triglyceride Transfer Protein	*MTP*	rs2306986	E98D (G>C)	0.030
		rs3816873	I128T (T>C)	0.266
		rs2306985	H297Q (C>G)	0.371
		rs17029215	D384A (A>C)	0.050
Niemann-Pick Disease, type C1	*NPC1*	rs1617407	5'UTR (G>A)	0.425
		rs1805081	H215R (T>C)	0.420
		rs1788799	M642I (G>C)	0.330
		rs1805082	I858V (T>C)	0.480

		rs1805084	R1266Q (C>T)	0.027
		rs1652376	3'UTR (G>T)	0.462
Nuclear receptor subfamily 1, group I, member 2	*NR1I2*	rs3814055	5'UTR (C>T)	0.385
		rs1523127	5'UTR (A>C)	0.399
Phosphatidylethanolamine N-methyltransferase	*PEMT*	rs7946	M175V (T>C)	0.247
Paraoxonase 1	*PON1*	rs854560	M55L (A>T)	0.362
		rs662	Q192R (A>G)	0.312
Paraoxonase 2	*PON2*	rs7493	C311S (G>C)	0.228
		rs12026	G148A (G>C)	0.228
Peroxisome Proliferator Activated Receptor α	*PPARA*	rs135539	-35,089A>C	0.452
		rs1800206	L162V (C>G)	0.063
		rs600825	3'UTR (G>A)	0.173
Peroxisome Proliferator Activated Receptor δ	*PPARD*	rs2038068	-68,915C>T	0.327
		rs2016520	-68,496G>A	0.211
Peroxisome Proliferator Activated Receptor γ	*PPARG*	rs1801282	25,506C>T	0.103
		rs2028759	P12A (C>G)	0.476
		rs3856806	161C>T	0.110
Scavenger Receptor Class B, member 1	*SCARB1*	Novel	-1254 G>A	0.038
		rs4238001	G2S (G>A)	0.098
		rs838884	3' UTR (A>G)	0.313
Sphingomyelin phosphodiesterase 1,acid lysosomal	*SMPD1*	rs1050228	A36V (G>A)	0.407
		rs1050239	G508R (G>A)	0.235
Srebp cleavage activating Protein	*SCAP*	rs12487736	I796V (A>G)	0.453
Sterol regulatory element binding protein 1a	*SREBF1a*	rs60282872	-36delG	0.152
Sterol O-acyltransferase 2	*SOAT2*	rs9658625	Q14G (A>G)	0.071
		rs2272296	T254I (C>T)	0.235
Transcription factor 1, hepatic;LF-B1, hepatic nuclear factor (HNF1)	*TCF1*	rs1169288	I27L (A>C)	0.354
		rs2464196	S487N (G>A)	0.318
		rs1169305	G573S (G>A)	0.001

Abbreviations: SNP: Single nucleotide polymorphism; MAF: Minor allele frequency. For non-synonymous SNPs, the change in amino acid and codon position are provided along with the nucleotide change. For SNPs in the 5' or 3' untranslated regions (UTR), the nucleotide change and the position in reference to the first nucleotide of initiation codon is shown, whenever known.
